# Incidence and risk factors for unplanned readmission after colorectal surgery: A meta-analysis

**DOI:** 10.1371/journal.pone.0293806

**Published:** 2023-11-16

**Authors:** Joel D’Souza, Simon Richards, Timothy Eglinton, Frank Frizelle

**Affiliations:** Department of Surgery, Christchurch Hospital, University of Otago, Dunedin, New Zealand; E-Da Cancer Hospital, TAIWAN

## Abstract

**Background:**

Unplanned readmissions (URs) after colorectal surgery (CRS) are common, expensive, and result from failure to progress in postoperative recovery. These are considered preventable, although the true extent is yet to be defined. In addition, their successful prediction remains elusive due to significant heterogeneity in this field of research. This systematic review and meta-analysis of observational studies aimed to identify the clinically relevant predictors of UR after colorectal surgery.

**Methods:**

A systematic review was conducted using indexed sources (The Cochrane Database of Systematic Reviews, MEDLINE, and Embase) to search for published studies in English between 1996 and 2022. The search strategy returned 625 studies for screening of which, 150 were duplicates, and 305 were excluded for irrelevance. An additional 150 studies were excluded based on methodology and definition criteria. Twenty studies met the inclusion criteria and for the meta-analysis. Independent meta-extraction was conducted by multiple reviewers (JD & SR) in accordance with PRISMA guidelines. The primary outcome was defined as UR within 30 days of index discharge after colorectal surgery. Data were pooled using a random-effects model. Risk of bias was assessed using the Quality in Prognosis Studies tool.

**Results:**

The reported 30-day UR rate ranged from 6% to 22.8%. Increased comorbidity was the strongest preoperative risk factor for UR (OR 1.39, 95% CI 1.28–1.51). Stoma formation was the strongest operative risk factor (OR 1.54, 95% CI 1.38–1.72). The occurrence of postoperative complications was the strongest postoperative and overall risk factor for UR (OR 3.03, 95% CI 1.21–7.61).

**Conclusions:**

Increased comorbidity, stoma formation, and postoperative complications are clinically relevant predictors of UR after CRS. These risk factors are readily identifiable before discharge and serve as clinically relevant targets for readmission risk-reducing strategies. Successful readmission prediction may facilitate the efficient allocation of healthcare resources.

## Introduction

The readmission rate is a key performance indicator that is widely used to evaluate the quality of hospital care delivered to a population [1–3]. Its well-validated association with the quality of healthcare, ease of calculation, and applicability to various patient cohorts underscores its clinical utility [1].

Unplanned readmissions (URs) have implications for both the patient and the healthcare system [4,5]. They result from a failure in the continued progression of patient recovery toward their preoperative baseline. This is supported by their association with poor patient outcomes such as increased morbidity, poor quality of life, and increased mortality [4,6]. URs are also associated with significant costs to the healthcare system, with an estimated economic impact of $17.4 billion per year in the US [7,8]. Their prevention is an important consideration for the rationing of limited healthcare resources in the Covid-19 era.

Previous research has highlighted fundamental differences between medical and surgical readmissions [7,8]. Medical patients comprised a higher proportion of overall readmissions and were readmitted for exacerbations of the underlying comorbidities. In contrast, most surgical patients were readmitted for complications related to the index surgery rather than exacerbations of underlying comorbidities. Therefore, surgical readmissions are more amenable to risk reduction interventions. When classified according to surgical specialty, colorectal surgery (CRS) has one of the highest readmission rates (25%) [7,9]. Successful readmission reduction interventions may therefore have a high yield in this specialty [6,9–20].

Prediction models derived from identified risk factors for readmission may be targeted toward high-risk individuals at discharge. If successful, this may facilitate the efficient use of transitional care strategies aimed at readmission reductio. A broad range of risk factors for UR after CRS have been reported in the literature. Due to limitations from significant heterogeneity in this field of research, this has not translated into effective readmission prediction [8,21–23]. The major sources of heterogeneity included variations in sample size, sample sources, methodology, definitions, time intervals to readmission, and non-standardized categorization of both demographic and clinical risk factors.

This systematic review and meta-analysis aimed to define the incidence of UR after CRS, identify the risk factors for UR derived from multivariable logistic regression, and further characterize the causes of UR. The heterogeneity observed in the literature was addressed by adherence to appropriate reporting standards. Risk factors were categorized clinically to create a foundation for future research.

## Methods

This systematic review and meta-analysis was conducted according to the Preferred Reporting Items for Systematic Review and Meta-Analyses (PRISMA) guidelines [24] and adapted to prognostic factor studies [25].

The question of this study was formulated according to the PICO strategy [26] as follows:

Population: Adult patients undergoing all types of colorectal surgery.
Intervention/Prognostic Factor: Reported prognostic/risk factors for UR.
Control/Comparison: No standard comparison was considered in this study.
Outcome: UR rate, risk factors, and readmission characteristics.

### Search strategy

The Cochrane Database of Systematic Reviews, MEDLINE, and EMBASE databases were searched upon consultation with a librarian using a combination of medical subject headings (MeSH) and key terms for full-text articles published between January 1996 and December 2022. The MeSH terms included “colorectal surgery,” and “patient readmission,”. Key terms included colorectal surgery, readmission, risk factors, predictors, and prediction. No publication language restrictions were applied to the search strategy. The full search strategy is available in the supplementary appendix (S1 Table in S1 Appendix).

### Study inclusion and exclusion criteria

The eligibility criteria was structured to define the UR rate and identify corresponding risk factors in patients who underwent all types of colorectal surgery. The study inclusion criteria was defined as adherence to all three of the following conditions: studies that evaluated UR as a primary outcome after colorectal surgery; studies that used a multivariable regression model to assess clinical variables for the primary outcome; and studies that reported the adjusted magnitude of association between the corresponding clinical variables and the primary outcome through odds ratios and corresponding 95% confidence intervals. Studies that included planned readmissions or patients directly discharged from the Emergency Department in the primary outcome were excluded. Studies that did not define the time-interval between the primary outcome and the index surgery were also excluded.

### Primary outcome and predictor variables

The primary outcome was defined as any unplanned readmission to the hospital within 30 days of index discharge. The predictor variables were defined according to the reported risk factors in the studies that met the inclusion criteria.

### Data extraction and quality assessment

The titles and abstracts of the identified studies were screened by two independent reviewers (JD and SR) to determine study eligibility according to criteria. Full-text papers were obtained and reviewed for all studies that could not be excluded by screening the title and abstract. The reference lists of the identified studies were manually searched for additional articles of relevance. Data were extracted from eligible studies by the same reviewers in accordance with the CHARMS-PF checklist [25]. These included the type of data, population and setting, sample size, colorectal surgery types, surgical indications, and types of predictor variables/ risk factors assessed using multivariable analysis. The methodological quality of the eligible studies was assessed using the Quality in Prognosis Studies (QUIPS) checklist [25]. Any discrepancy in methodological quality between the primary reviewers was resolved through discussion with a third reviewer (TE and FF).

### Statistical analysis

Descriptive statistics were obtained from the included studies. All clinical variables and their corresponding ORs and 95% Confidence Intervals (CI) were tabulated. Comparable clinical variables, where applicable, were grouped and displayed on forest plots to highlight their frequency of assessment and the variability in the magnitude of association with the primary outcome. Given the expected heterogeneity in prognostic factor research, pooled estimates with 95% CIs were calculated through a meta-analysis for comparable variables using a random-effects model. Heterogeneity was expressed using the I^2^ statistic. I^2^ is the percentage of variability in effect size, which is not attributable to sampling errors. Threshold I^2^ values of 25%, 50%, and 75% were used to define low, moderate, and high heterogeneity, respectively. Meta-regression was performed for the pooled estimates of variables with more than ten studies. Statistical analysis was performed using the “dmetar” package in RStudio (version 4.2.2) [27].

## Results

### Search results

**Fig 1:**
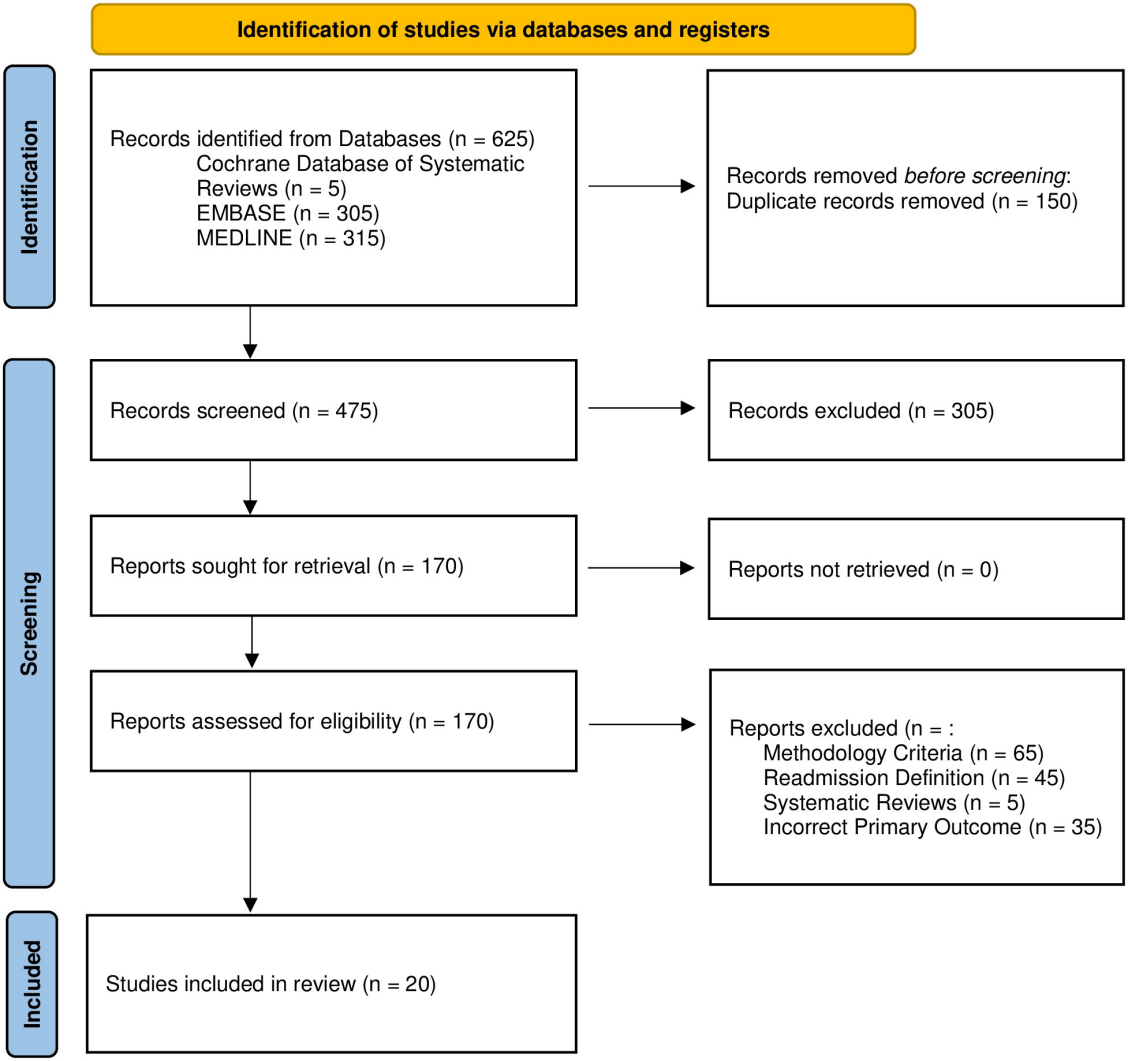
PRISMA flow diagram.

The initial database search using our strategy returned 625 studies. The duplicates were removed (n = 150) and the remaining 475 titles and abstracts screened. Three hundred and five citations were excluded based on conference abstracts and relevance. Full texts were retrieved for the remaining 170 citations, and 150 articles were subsequently excluded based on the described methodology criteria, readmission definition, and primary outcome. The literature cited in these articles was reviewed and no further relevant articles were identified. Twenty articles were included in this systematic review [6,13–15,28–43]. The search results and the selection process are summarized in Fig 1.

### Study characteristics

The data extracted from the included studies are presented in Tables 1 and 2. All the studies were retrospective and observational in their design. The study sample sources included national databases (n = 9), multi-institution databases (n = 4), single-institution databases (n = 6), and an insurance claims database (n = 1). The sample sources between studies were independent and the sample sizes ranged from 368 to 3367921 patients. The number of corresponding predictor variables evaluated ranged from seven to twenty-five. These were further classified into preoperative, perioperative, and postoperative variables. The evaluated colorectal procedures included colonic resections, rectal resections, and stoma surgery for both benign and malignant indications. Seven studies had a moderate-to-high risk of bias due to sample sources, sample size, and prognostic factor characteristics as presented in S2 Table in S1 Appendix.

**Table 1:** Study characteristics.

Study	Year	Country	Study Design	Data Source	Study period	Consecutive recruitment	Setting	Sample Size	Mortality Exclusions
Almussallam	2016	USA	Retrospective	Single Institution	2007–2011	Yes	Not Specified	4879	Yes
Barina	2020	Italy	Retrospective	National Dataset	2002–2014	Not Specified	Acute & Elective	47704	Yes
Bliss	2015	USA	Retrospective	Multiple Institution Administrative Dataset	2007–2011	Not Specified	Acute & Elective	93913	Yes
Damle	2014	USA	Retrospective	Multiple Institution	2008–2011	Not Specified	Acute & Elective	70484	Yes
Devon	2011	Canada	Retrospective	Multiple Institution	1996–2004	Not Specified	Acute & Elective	12415	Yes
Frances	2015	UK	Retrospective	Single Institution	2002–2009	Yes	Elective only	268	Yes
Ghirimoldi	2020	USA	Retrospective	Single Institution	2013–2017	Yes	Acute & Elective	677	Not Specified
Greenblatt	2010	USA	Retrospective	National Database (SEER)	1992–2002	Not Specified	Acute & Elective	42348	Yes
Kariv	2006	USA	Retrospective	Single Institution	2003–2004	Not Specified	Acute & Elective	1179	Yes
Keller	2014	USA	Retrospective	Single Institution	2006–2012	Not Specified	Acute & Elective	3504	Yes
Kulaylat	2015	USA	Retrospective	Multiple Institution	2011	Not Specified	Acute & Elective	10155	Not Specified
Lucas	2014	USA	Retrospective	National Database (SEER)	1997–2002	Not Specified	Not Specified	44822	Not Specified
Lumpkin	2018	USA	Retrospective	National Administrative Database (NRD)	2010–2014	Not Specified	Acute & Elective	3367921	Yes
Pucciarelli	2017	Italy	Retrospective	National Database	2005–2014	Not Specified	Acute & Elective	345074	Yes
Rattan	2018	USA	Retrospective	National Administrative Database (NRD)	2013–2014	Not Specified	Acute & Elective	79098	Yes
Schneider	2012	USA	Retrospective	National Database (SEER)	1986–2005	Not Specified	Acute & Elective	143386	Yes
Sutton	2014	USA	Retrospective	National Database (UHC)	2009–2012	Not Specified	Not Specified	4952	Yes
Toneva	2013	USA	Retrospective	National Database (VASQIP)	2005–2009	Not Specified	Elective only	8180	Yes
Turina	2013	USA	Retrospective	Single Institution	2010–2011	Not Specified	Not Specified	564	Yes
Wick	2011	USA	Retrospective	Multiple Institution Insurance Claims Database	2002–2008	Not Specified	Not Specified	10882	Not Specified

**Table 2:** Study variable characteristics.

Study	Year	Total Variables	Preoperative Variables	Operative Variables	Postoperative Variables	Readmissions	Readmission Rate (%)	Events per Predictor Variable
Almussallam	2016	17	9	6	2	492	10.1	29
Barina	2020	9	7	2	0	2122	4.4%	236
Bliss	2015	14	9	3	2	13955	14.9	997
Damle	2014	17	8	3	6	9632	13.7	567
Devon	2011	11	9	1	1	1898	15.3	173
Frances	2015	7	1	0	6	34	12.7	5
Ghirimoldi	2020	4	2	1	1	155	22.9	39
Greenblatt	2010	25	18	2	5	4662	11.0	186
Kariv	2006	32	9	8	15	150	12.7	5
Keller	2014	7	4	1	2	212	6.1	30
Kulaylat	2015	13	7	5	1	1492	14.7	115
Lucas	2014	18	17	1	0	5502	12.3	306
Lumpkin	2018	9	9	0	0	43546	13.0	4838
Pucciarelli	2017	14	9	3	2	20808	6.0	1486
Rattan	2018	16	11	3	2	5591	7.1	349
Schneider	2012	8	4	1	3	16753	11.7	2094
Sutton	2014	5	5	0	0	1131	22.8	226
Toneva	2013	7	4	3	0	1161	14.2	166
Turina	2013	6	3	2	1	105	18.6	18
Wick	2011	11	5	2	4	1239	11.4	113

### Primary outcome: Unplanned readmission rate and diagnoses

**Fig 2:**
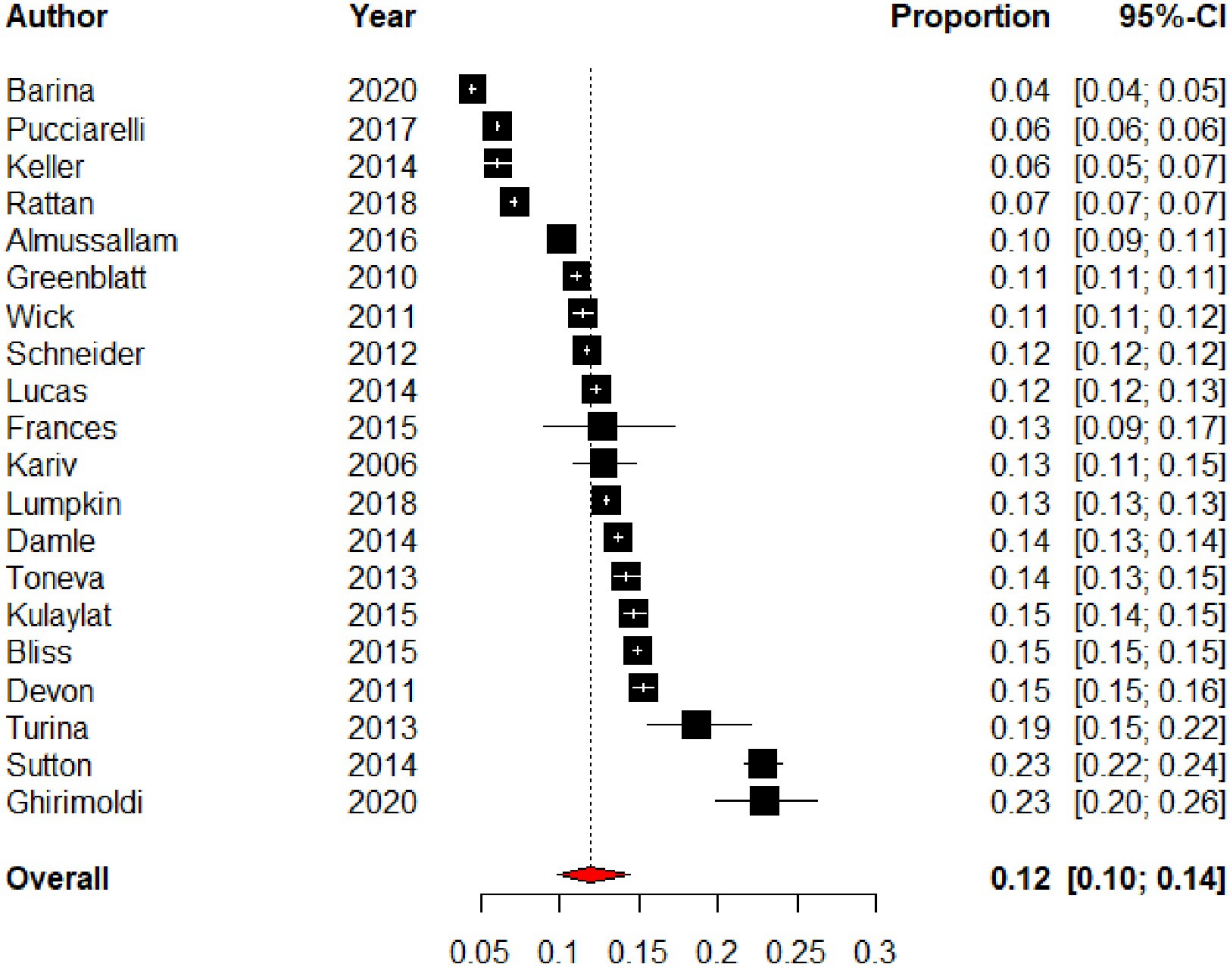
Forest plot of unplanned readmission rate.

The median UR rate reported in the included studies was 12.7% (IQR:11.2–14.8). The pooled UR rate was 12% (95% CI 10%—14%) as shown in Fig 2. The three most common reasons for UR in each study are presented in Table 3. Surgery-related infections and gastrointestinal complications were the most common reasons for readmission. Only four studies reported the median time to readmission from index discharge. This ranged from seven days to thirteen days.

**Table 3:** Reasons for unplanned readmission.

Study	Reasons for Readmission	Timing (median + IQR)
Almussallam	Not Reported	Not Reported
Barina	Not Reported	Not Reported
Bliss	Infections 32.5% Gastrointestinal (GI)/Malnutrition 16.8% Ileus: 7.4%	Not Reported
Damle	Gastrointestinal 29% Surgical site related 28% Other: 20%	7 days
Devon	Not Reported	Not Reported
Frances	Wound Infection: n = 6 Ileus: n = 6 Bowel Obstruction: n = 4	Not Reported
Ghirimoldi	Not Reported	Not Reported
Greenblatt	Ileus/obstruction/GI: 28.3% Surgical site infection (SSI): 7.6% Pneumonia/Respiratory complications: 7.1%	Median: 9 (4–17)
Kariv	Surgical Site infection (SSI): n = 55 Ileus/Small Bowel Obstruction: n = 39 Medical Complications: n = 41	10 days +/- 8.2
Keller	Dehydration	Not Reported
Kulaylat	Not Reported	Not Reported
Lucas	Not Reported	Not Reported
Lumpkin	Infection 5% GI Complications/Malnutrition 3% Electrolyte Disorder/fluid imbalance	Not Reported
Pucciarelli	Infections 19% Obstruction 14.6% Urinary tract infection 9.1%	Not Reported
Rattan	Infection 14.3% GI device malfunction 11.7% Intestinal obstruction	Not Reported
Schneider	Operative complications (21.8%) Dehydration (21.5%) Postoperative infection (18.3%)	Not Reported
Sutton	Not Reported	Not Reported
Toneva	Not Reported	Not Reported
Turina	Ileus 4.8% SSI 4.6% Reoperation 4.4%	Not Reported
Wick	Gastrointestinal 56.1% SSI 47.1% Reoperation: 24.5%	13.3 days

### Risk factors for 30-day unplanned readmission

The frequency of all variables examined in the included studies are shown in Tables 4 and 5 Preoperative variables were the predominant variable types evaluated (n = 34). The variables ranged from clinical to hospital-system level variables.

**Table 4:** All preoperative variables.

Variable Characteristic	Number of studies that assessed the variable	Number of studies where the variable was significant on multivariable analysis
Cumulative Comorbidity	15	12
Age	15	9
Sex	14	8
Surgical Urgency	10	6
Hospital Volume	7	3
Insurance Provider	5	5
Race	6	5
Year of Treatment	5	2
Cohabitation/functional status	3	2
Income/Socioeconomic status	4	3
Individual comorbidity	3	3
Previous abdominal surgery	3	1
Hospital bed Size	2	1
Hospital geographical location	2	1
Hospitalisation year before surgery	3	3
Hospital setting: rural vs metropolitan	2	1
Hospital teaching status	2	2
Alcohol use	1	1
Anxiolytic use	1	1
BMI	1	0
Hospital ownership	1	0
Intestinal obstruction/perforation	1	0
Marital Status	1	0
Obesity	1	0
Patient setting	1	1
Preoperative Steroids	1	1
Preoperative treatment	1	1
Previous Anticoagulation	1	1
Smoking	1	0
Stage of disease	1	0
Surgeon Volume	2	1
Transferred for surgery	2	0
Tumour Grade	1	1
Surgery 3 years prior to surgery	2	0

**Table 5:** All operative and postoperative variables.

Variable Characteristic	Number of studies that assessed the variable	Number of studies where the variable remained significant on multivariable analysis
**Operative Variables**		
Stoma	12	11
Surgical Approach	8	4
Surgical Urgency	10	5
Indication	8	8
Procedure Type	7	7
Rectal procedure	3	3
Abscess	1	0
Presence of Anastomosis	1	0
Bowel perforation	1	1
Bowel prep	1	1
Estimated Blood Loss	1	0
Metastasis	1	1
Mode of Anaesthesia	1	0
Obstruction	1	1
Peri-operative steroids	1	1
Stoma Type	1	0
Surgery time (per 30 minutes)	1	1
Anastomosis Type	1	0
Wound Class	1	0
**Postoperative Variables**		
Length of Stay (days)	8	7
Discharge Disposition	8	4
Any Postoperative Complications	7	7
Specific postoperative Complications		
Blood transfusion	2	2
ICU Admission	2	2
Postoperative Ileus	2	0
Reoperation	2	1
Epidural anaesthetic failure	1	0
Interventional drainage	1	1
Surgical site infection	1	1
Antibiotics on Discharge	1	0
Chemotherapy within 30 days of discharge	1	0
Discontinuation of intravenous fluids	1	0
Emergency Department visit	1	0
ERAS Compliance	1	1
ERAS Deviation	1	1
Biochemical laboratory tests	1	0
Steroids on Discharge	1	1

### Preoperative variables: Age, sex, and comorbidity

Age was one of the most common variables in the included studies (n = 15). On multivariable analysis, it was analyzed as a continuous variable in one study, and a categorical variable in the remaining 14 studies. The threshold for categorization and the corresponding reference group varied significantly between studies, thus limiting further pooled analysis.

Sex was evaluated in 14 studies and was a significant variable in 8 studies. It was not a statistically significant risk factor of UR in the pooled analysis (OR 0.96, 95% CI 0.90–1.02), S1 Fig in S1 Appendix.

**Fig 3:**
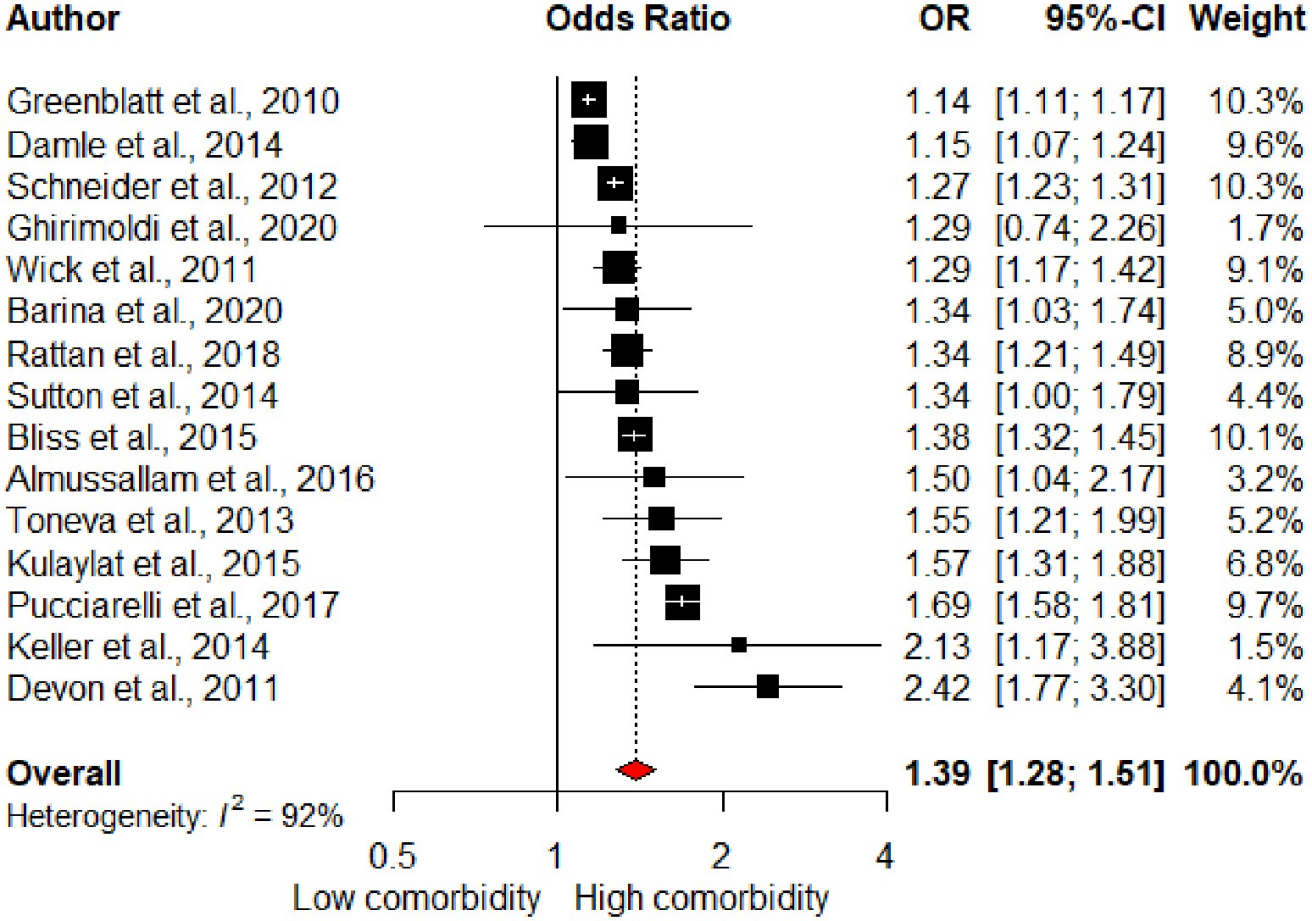
Forest plot of comorbidity and unplanned readmission. *Kariv et al., 2006 excluded from the forest plot as non-significant OR and 95%CI not reported.

Sixteen studies assessed cumulative comorbidity using various instruments. These included the American Society of Anesthesiologists (ASA) score, Charlson Comorbidity Index (CCI), Elixhauser score, Hierarchical Condition Categories (HCC), Risk Analysis Index-A (RAI-A), and Severity of Illness (SOI) score. Due to the variability in the instruments, the reported OR from the highest comorbidity category in relation to the lowest “reference” category of each instrument was used. Increased comorbidity was a significant risk factor for UR in the pooled analysis (OR 1.39, 95% CI 1.28–1.51), Fig 3. Some studies had evaluated individual conditions as risk factors, but the inconsistency between these studies limited further comparison.

### Operative variables: Surgical indication, acuity, approach, and stoma formation

Surgical indication was a significant variable in all seven studies that evaluated it. These studies included a wide range of both benign and malignant indications, thus limiting pooled analysis. Malignancy, diverticulitis, and inflammatory bowel disease (IBD) were the most common indications. Similarly, surgery type was significant overall but varying reference levels between studies limited further comparison.

**Fig 4:**
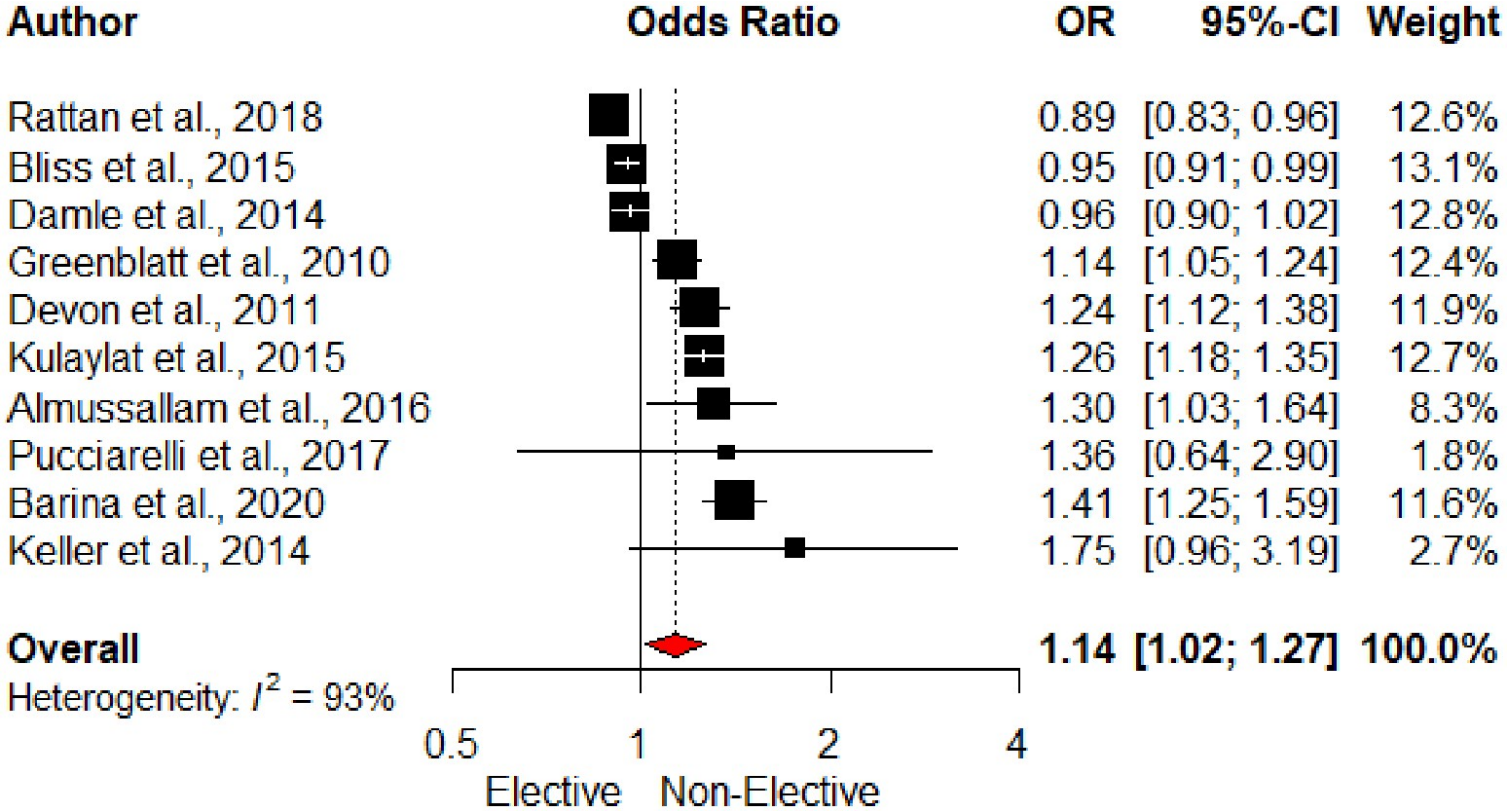
Forest plot of surgical acuity and unplanned readmission. *Kariv et al., 2006 excluded from the forest plot as non-significant OR and 95%CI not reported.

Surgical acuity was assessed in eleven studies and was significant variable in 6 studies. Non-elective surgery was a significant risk factor for UR on pooled analysis (OR 1.14, 95% CI1.02–1.27), Fig 4.

Surgical approach was evaluated in eight studies. Although some studies included robotic surgery, the derived effect size was limited to the laparoscopic and open approaches (reference). The evidence was conflicting, resulting in a non-significant pooled effect size (OR 0.90, 95% CI 0.81–1.01), S2 Fig in S1 Appendix.

**Fig 5:**
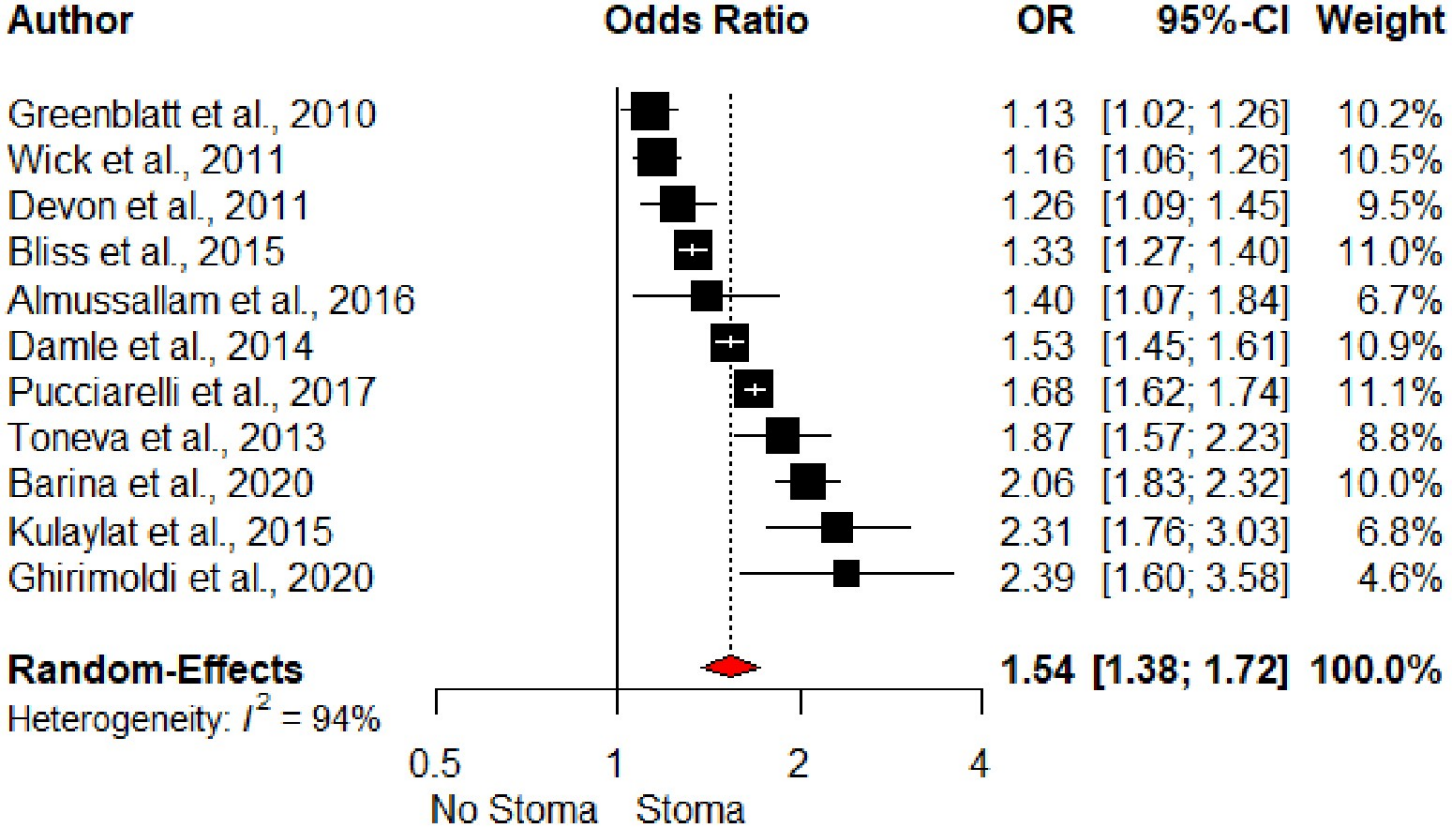
Forest plot of stoma and unplanned readmission. *Kariv et al., 2006 excluded from the forest plot as non-significant OR and 95%CI not reported.

Stoma formation was the most common operative variable in the included studies (n = 12). It was a significant variable in eleven out of the twelve studies, and the strongest operative risk factor for UR in the pooled analysis (OR 1.54, 95% CI 1.38–1.72), Fig 5).

### Postoperative variables: Discharge disposition and postoperative complications (PoC)

**Fig 6:**
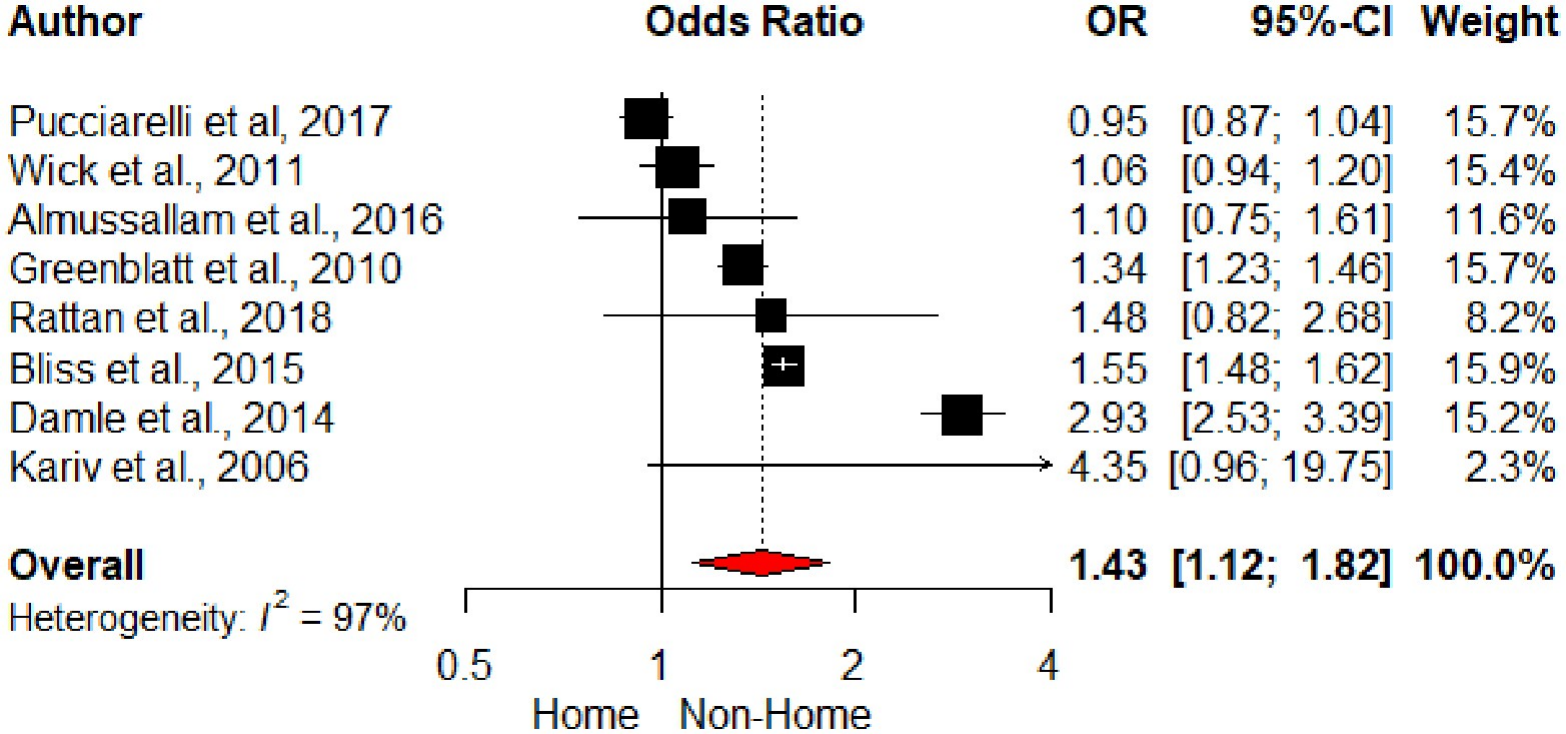
Forest plot of discharge disposition and unplanned readmission.

Discharge disposition was significant risk factor for UR on pooled analysis (OR 1.42, 95% CI 1.12–1.82;), Fig 6. The classification of discharge disposition varied across studies. Home was used as the reference level and the effect size was pooled for non-home discharge. This variable included discharge to rehabilitation and short-term nursing facility.

**Fig 7:**
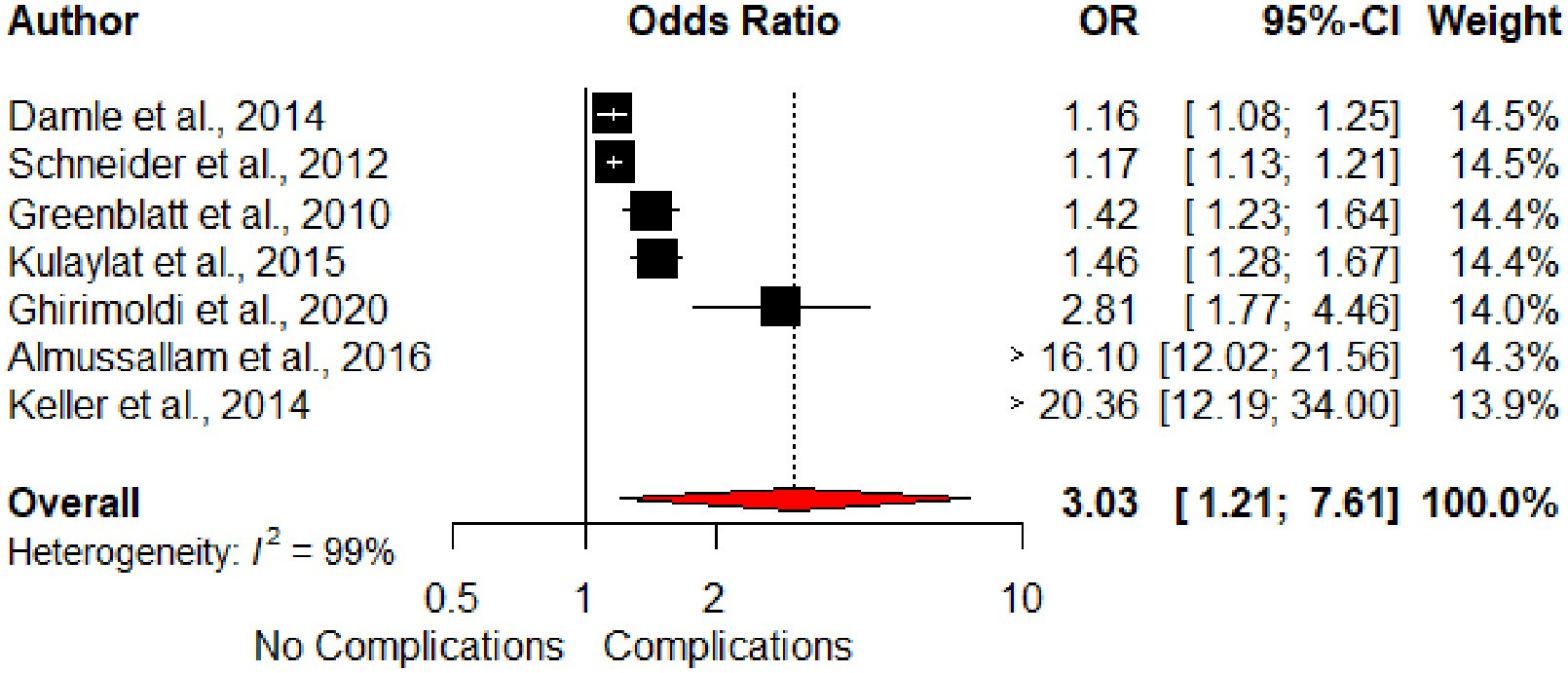
Forest plot of postoperative complications and unplanned readmission.

The occurrence of postoperative complications was evaluated in seven studies. It was the strongest postoperative risk factor for UR on pooled analysis (Fig 7). Patients who developed a PoC were more likely to be readmitted than those who did not develop a PoC (pooled OR 3.03, 95% CI 1.21–7.61).

## Discussion

The findings of this systematic review and meta-analysis demonstrate that URs are common, and on average, 12.7% of CRS procedures result in UR within 30 days of the index discharge. Although inconsistently reported, the most common readmission diagnoses were PoCs related to the index surgery. These findings support the use of UR as a KPI for colorectal surgery.

This study identified multiple risk factors for UR. As expected, their definitions, modifiability, frequency of assessment, threshold of significance, and magnitude of association with UR varied. However, certain risk factors were consistently associated with UR and their categorization into preoperative, operative, and postoperative phases of surgical care unveiled their clinical utility.

Increased comorbidity was a significant preoperative risk factor for UR. Additional studies both within and outside the scope of this review reported similar findings [5,20,23,44,45]. Multiple instruments were used to measure comorbidity in the included studies with the lowest comorbidity category of each instrument used as the reference level. To facilitate comparison between instruments, the reported OR from the highest comorbidity category relative to the reference category within each instrument was used. The association between comorbidity and UR remained significant despite the resultant heterogeneity from the variation in instrument use and categorization. Further homogenous research through standardised instrument use is required for the accurate quantification of effect size. It is likely that this association overlaps with the well-supported association between increased comorbidity and the occurrence of PoCs [9]. The extent of this overlap is difficult to delineate as only six of the 14 studies in this review that assessed comorbidity also controlled for the variable of PoCs in their respective multivariable analysis.

The preoperative identification of patients with increased comorbidities serves two purposes in the readmission context. First, these patients may be targeted with readmission reduction interventions, given their high risk of UR. Second, it may provide an opportunity for prehabilitation and medical optimization to reduce surgical morbidity.

Stoma formation was the strongest operative risk factor for UR in this study. This is consistent with the findings of other systematic reviews on colorectal surgery readmissions [21,22]. Their utility in the management of septic complications of anastomotic leaks is accompanied by challenges in patient adjustment, electrolyte imbalances, and renal failure. Stomas are clearly indicated in certain scenarios and stoma-related complications are often of lower severity. In the readmission context, they provide another clinically useful target for identifying patients at high risk of readmission.

The development of PoCs was the strongest postoperative and overall risk factor for UR (OR 3.03, 95% CI 1.21–7.61). Multiple studies across a range of surgical specialties have also reported similar findings to support this association [4,9,20,45–47]. In addition, a dose-response relationship between the number of PoCs and the risk of UR has been reported in studies outside of colorectal surgery [47–51].

Given that the most common reasons for UR, when reported, were PoCs related to the index surgery, it is likely that URs in the CRS setting are surrogates for PoCs that develop after discharge. However, this is difficult to delineate based on the studies included in this systematic review because of several limitations. Firstly, clinical characteristics of PoCs were not reported in most studies. Secondly, the true proportion of URs for PoCs could not be ascertained due to significant variation in reported proportions between studies and the complete absence of reporting in the remaining studies. Thirdly, it was unclear whether the PoCs that resulted in UR were clinically apparent before discharge. Finally, it was also unclear whether the PoCs that led to UR were related to any PoC before discharge or if they were completely new PoCs that developed after discharge. Further research to address these limitations may delineate how the number, severity, and timing of PoCs may modulate the risk of URs after colorectal surgery. It may also indicate whether these URs are reducible, if not preventable, through measures aimed at reducing the incidence of PoCs.

Discharge disposition was a significant postoperative risk factor for UR, but the underlying aetiology was unable to be delineated from the included studies. Pertinent aspects may include poor communication with the nursing facility, low inherent threshold of the facility to transfer back to the index hospital, or the development of complications. Further research is required to ascertain its role as a supplementary target for readmission-reduction interventions.

The studies included in this systematic review and meta-analysis have highlighted the wide range of risk factors reported in the literature. The full array of comparable and non-comparable risk factors that were evaluated in both individual and multiple studies that met the eligibility criteria, are presented in S3-S7 Tables in S1 Appendix. Clinical significance varied between each risk factor and was further lost in the multiplicity when combined with inconsistent reporting standards and non-standardised categorisation. As an example, Length of stay (LOS) was a significant postoperative variable in seven of the eight studies, but pooled analysis was limited due to the varied threshold of significance. In addition, LOS as a variable is affected by multiple factors, including patient recovery time, postoperative complications, hospital resources, and discharge facility resources. This risk factor was therefore of limited clinical utility and a significant contributor to the observed heterogeneity. Future research in this field requires an emphasis on clinically significant risk factors with standardised definitions and categorisation.

Further known clinically relevant risk modifying factors such as ERAS adherence, the use of biologics, and further delineation of stoma types were not reported in the literature. In the context of Enhanced Recovery after Surgery (ERAS), previous meta-analyses have demonstrated that ERAS does not affect readmission rates [52].

Transitional care interventions (TCIs) aimed at reducing medical readmissions include discharge planning, medical reconciliation, patient education, follow-up telephone calls, and timely primary specialist care follow-up [5,53–56]. None of these interventions were associated with reduced readmission rates when implemented alone [53]. The efficacy of these interventions in surgical patients remain unclear given the differences in clinical needs of surgical patients. TCIs are labor intensive and expensive. If found to be effective in colorectal surgery patients, reliable readmission prediction may facilitate targeted TCIs to maximize the efficiency of this invaluable resource.

A recent systematic review of risk prediction models for medical readmissions concluded that improvements in predictive model performance require further research on social support as a potential predictor of readmission [2]. Similarly, the impact of frailty on adverse postoperative outcomes, including UR, has recently been highlighted across a range of surgical specialties [57]. The influence of these two potential risk factors on UR remains a gap in our current understanding.

### Limitations

There are certain limitations that should be considered when interpreting the results of this study. The nature of prognostic factor research is accompanied by heterogeneity [25]. This study was conducted to delineate trends and identify risk factors for UR from existing literature, considering this heterogeneity.

Despite the strict eligibility criteria to minimise this, the retrospective study design, sample source variation, and the non-standardized classification and categorization of prognostic factors resulted in significant heterogeneity, as represented by the I^2^ value of the forest plots. We also acknowledge limitations in the delineation of how each risk factor in this meta-analysis correlated with each other due to the discrepancy in covariates of each multivariable model between studies.

Meta-regression was performed for cumulative comorbidity and stoma variables as they were evaluated in more than 10 studies (S3 Fig in S1 Appendix). There was no evidence that risk of bias was associated with the effect size of both stoma and cumulative comorbidity variables. Visual inspection and further interpretation of the funnel plot presented S4 Fig in S1 Appendix was limited by between-study heterogeneity (Egger’s test, p = 0.84).

## Conclusions

Patients with increased comorbidities, stoma formation, and those who developed PoCs were at an increased risk of UR. As a clinical tool, these variables are easy to identify before discharge, and may serve as useful targets for potential interventions. Further research is required to scrutinize these initial findings, given the significant heterogeneity encountered in this subject. Additional risk factors, both novel and those that may be currently masked by this heterogeneity, may be identified through this process.

## Supporting information

S1 Checklist PRISMA checklist. (DOCX)

S1 Appendix Supplementary appendix. (DOCX)

S1 File Risk of bias. (CSV)

S2 File Cumulative comorbidity. (CSV)

S3 File Discharge disposition. (R)

S4 File Proportions. (CSV)

S5 File Postoperative complications. (CSV)

S6 File Sex. (CSV)

S7 File Stoma. (CSV)

S8 File Surgical approach. (CSV)

S9 File Surgical urgency. (CSV)

S10 File Meta analysis R Code. (CSV)
