# Supplementary material for: Incidence and risk factors for unplanned readmission after colorectal surgery: A meta-analysis
Source: PLoS One. 2023 Nov 16;18(11):e0293806. doi: 10.1371/journal.pone.0293806 (PMC10653493; doi:10.1371/journal.pone.0293806)
Supplement: S1 Appendix — (DOCX) [file pone.0293806.s002.docx]

# Title: Incidence and risk factors for unplanned readmission after colorectal surgery: a meta-analysis

Authors

Joel D’Souza^1*^, Simon Richards^1^, Timothy Eglinton^1^, Frank Frizelle^1^

^1^Department of Surgery, Christchurch Hospital.

*Corresponding author

[joelds541@gmail.com](mailto:joelds541@gmail.com) (JD)

**ORCID ID: 0000-0003-2570-6336**

**Supplementary Materials Index**

**Supplementary Methods:** Nil

**Supplementary Results:** Nil

**Supplementary Results:** Nil

**Supplementary Appendixes:** Nil

**Supplementary Tables**

S1 Table: Search strategy.

S2 Table: Risk of bias.

S3 Table: Risk factors assessed in multiple studies.

S4 Table: Statistically significant preoperative variable (individual studies).

S5 Table: Statistically significant operative variables (individual studies).

S6 Table: Statistically significant postoperative variables (individual studies).

S7 Table: Statistically non-significant variables (individual studies).

**Supplementary Figures**

S1 Fig: Forest plot of sex and unplanned readmission.

S2 Fig: Forest plot of surgical approach and unplanned readmission

S3 Fig: Meta regression bubble plot.

S4 Fig: Funnel plot

S5 Fig: Risk of bias plot.

**References:** Nil

S1 Table: Search strategy

| 1. Colorectal Surgery.tw |
| --- |
| 2. Colorectal Surgery.mp |
| 3. 1 OR 2 |
| 4. Patient Readmission/ |
| 5. Readmission.tw |
| 6. Readmission.mp |
| 7. 4 OR 5 OR 6 |
| 8. Risk Factors.tw |
| 9. Risk Factors.mp |
| 10. 8 or 9 |
| 11. Predict$.tw |
| 12. Predict$.mp |
| 13. 11 OR 12 |
| 14. 10 OR 13 |
| 15. 3 AND 7 AND 14 |

S2 Table: Risk of bias

| **Study** | **Year** | **Study participation** | **Study Attrition** | **Prognostic Factor measurement** | **Outcome measurement** | **Adjustment of other prognostic factors** | **Statistical analysis and reporting** | **Overall** | **Reasons for moderate/high**  **heterogeneity** |
| --- | --- | --- | --- | --- | --- | --- | --- | --- | --- |
| Almussallam | 2016 | Low | Low | Low | Low | Moderate | Low | Low |  |
| Barina | 2020 | Low | Low | Low | Low | Moderate | Low | Low |  |
| Bliss | 2015 | Low | Moderate | Low | Low | Moderate | Low | Moderate | Administrative/cost database. |
| Damle | 2014 | Low | Low | Low | Low | Moderate | Low | Low |  |
| Devon | 2011 | Low | Low | Low | Low | Moderate | Low | Low |  |
| Frances | 2015 | Low | Low | Low | Low | Moderate | High | High | Inadequate sample size. |
| Ghirimoldi | 2020 | Low | Low | Low | Low | Moderate | Low | Low |  |
| Greenblatt | 2010 | Low | Low | Low | Low | Moderate | Low | Low |  |
| Kariv | 2006 | Low | Low | Low | Low | Moderate | High | High | Inadequate sample size. |
| Keller | 2014 | Low | Low | Low | Low | Moderate | Low | Low |  |
| Kulaylat | 2015 | Low | Low | Low | Low | Moderate | Low | Low | All-payer database. |
| Lucas | 2014 | Low | Low | Low | Low | Moderate | Low | Low |  |
| Lumpkin | 2018 | Low | Moderate | Low | Low | High | Low | High | No operative and postoperative variables. Administrative database. |
| Pucciarelli | 2017 | Low | Low | Low | Low | Moderate | Low | Low |  |
| Rattan | 2018 | Low | Low | Low | Low | Moderate | Low | Low | Administrative database. |
| Schneider | 2012 | Low | Low | Low | Low | Moderate | Low | Low |  |
| Sutton | 2014 | Low | Moderate | Low | Low | Moderate | Low | Moderate | Readmissions to other hospitals not included. |
| Toneva | 2013 | Low | High | Low | Low | Moderate | Low | High | Excluded Length of stay (LOS)> 30, n = 234. |
| Turina | 2013 | Low | Low | Low | Low | Moderate | Low | Low |  |
| Wick | 2011 | High | High | Low | Low | High | High | High | Insurance claims database. |

S3 Table: Risk factors assessed in multiple studies.

| **Variables** | **Author** | **Reference Level** | **Significance Level** | **Odd’s Ratio** | **Lower 95% CI** | **Upper 95% CI** |
| --- | --- | --- | --- | --- | --- | --- |
| **Preoperative variables** | | | | | | |
| **Age (years)** | Almussallam | >59 | 40 - 59 | 1.20 | 1.00 | 1.60 |
|  |  |  | <40 | 2.10 | 0.40 | 3.00 |
|  | Barina | 65 - 74 | 75 - 84 | 1.05 | 0.96 | 1.15 |
|  |  |  | 85+ | 1.10 | 0.92 | 1.31 |
|  | Bliss | >64 | <64 | 1.20 | 1.12 | 1.28 |
|  | Damle |  | per 10-year increase | 1.00 | 1.00 | 1.00 |
|  | Devon | >65 | 65 - 74 | 1.14 | 1.05 | 1.25 |
|  |  |  | 75 - 79 | 1.30 | 1.19 | 1.45 |
|  |  |  | 80+ | 1.59 | 1.44 | 1.75 |
|  | Greenblatt | 66 - 70 | 71 - 75 | 0.94 | 0.85 | 1.05 |
|  |  |  | 76 - 80 | 1.00 | 0.90 | 1.11 |
|  |  |  | 81 - 85 | 1.04 | 0.93 | 1.16 |
|  |  |  | 86+ | 1.08 | 0.96 | 1.21 |
|  | Kulaylat | <45 | 45 - 65 | 0.85 | 0.69 | 1.03 |
|  |  |  | 65 - 75 | 0.90 | 0.72 | 1.15 |
|  |  |  | 75+ | 1.13 | 1.90 | 1.40 |
|  | Lucas | 60 | 70 | 1.10 | 1.00 | 1.20 |
|  |  |  | 80 | 1.23 | 1.12 | 1.35 |
|  |  |  | >90 | 1.24 | 1.08 | 1.42 |
|  | Lumpkin | 35 - 49 | 18 - 34 | 1.04 | 0.99 | 1.10 |
|  |  |  | 50 - 64 | 0.92 | 0.89 | 0.95 |
|  |  |  | >65 | 0.77 | 0.74 | 1.80 |
|  | Pucciarelli | <50 | 50 - 59 | 0.89 | 0.83 | 0.96 |
|  |  |  | 60 - 69 | 0.89 | 0.83 | 0.95 |
|  |  |  | 70 - 79 | 0.94 | 0.88 | 1.01 |
|  |  |  | 80+ | 1.02 | 0.95 | 1.10 |
|  | Rattan | <50 | 51 - 60 | 0.87 | 0.80 | 0.95 |
|  |  |  | 61 - 69 | 0.76 | 0.69 | 0.84 |
|  |  |  | 70 - 90 | 0.75 | 0.66 | 0.84 |
|  | Schneider | <75 | 75+ | 1.05 | 1.01 | 1.09 |
|  | Sutton | 44 | 41 | 0.99 | 0.99 | 1.00 |
|  | Toneva | <65 | >65 | NS | NR | NR |
|  | Wick | <55 | >55 | 0.97 | 0.91 | 1.03 |
| **Cumulative comorbidity** | Almussallam | ASA: 2 - 3 | ASA: <2 | 0.90 | 0.60 | 1.30 |
|  |  |  | ASA: >3 | 1.10 | 0.80 | 1.50 |
|  |  | CCI: 0 | CCI: 1 - 3 | 1.10 | 0.80 | 1.40 |
|  |  |  | CCI: >3 | 1.50 | 1.00 | 2.10 |
|  | Barina | CCI: 0 | CCI: 1 - 2 | 1.37 | 1.23 | 1.52 |
|  |  |  | CCI: 3+ | 1.34 | 1.03 | 1.74 |
|  | Bliss | Elixhauser score: 0 - 1 | Elixhauser score: >2 | 1.38 | 1.32 | 1.45 |
|  | Damle | SOI: Low | SOI: High | 1.15 | 1.07 | 1.24 |
|  | Devon | CCI: 0 | CCI: 1 | 1.33 | 1.18 | 1.50 |
|  |  |  | CCI: 2 | 1.62 | 1.32 | 1.98 |
|  |  |  | CCI: 3+ | 2.42 | 1.77 | 3.30 |
|  | Ghirimoldi | RAI-A: Robust | Normal | 1.01 | 0.64 | 1.56 |
|  |  |  | Frail | 1.29 | 0.73 | 2.24 |
|  | Greenblatt | HCC: 2.1 | HCC 2.6 | 1.14 | 1.11 | 1.17 |
|  | Kariv | ASA: NR | ASA: NR | NS |  | 3.88 |
|  | Keller | ASA: NR | ASA: NR | 2.13 | 1.17 | 1.26 |
|  | Kulaylat | CCI: 0 | CCI: 1 | 1.03 | 0.86 | 1.24 |
|  |  |  | CCI: 2+ | 1.57 | 1.31 | 1.88 |
|  | Pucciarelli | CCI: 0 | CCI: 1 - 2 | 1.22 | 1.17 | 1.81 |
|  |  | CCI: 0 | CCI: 3+ | 1.69 | 1.58 | 1.49 |
|  | Rattan | CCI: <1 | CCI: >1 | 1.34 | 1.21 | 1.31 |
|  | Schneider | CCI: <3 | CCI: >3 | 1.27 | 1.23 | 1.31 |
|  | Sutton | SOI: Mild | SOI: Moderate | 1.17 | 0.86 | 1.41 |
|  |  |  | SOI: Severe | 1.49 | 0.98 | 1.90 |
|  |  |  | SOI: Extreme | 1.34 | 1.17 | 2.09 |
|  | Toneva | ASA: 1 - 2 | ASA: 3 | 1.13 | 0.96 | 1.33 |
|  |  |  | ASA: 4 - 5 | 1.55 | 1.21 | 2.00 |
|  | Wick | SOI: 1 - 2 | SOI: 3 | 1.13 | 1.05 | 1.21 |
|  |  |  | SOI: 4 | 1.29 | 1.17 | 1.42 |
| **Previous abdominal surgery** | Kariv | No | Yes | NR | NR | NR |
|  | Keller |  |  | 1.62 | 0.99 | 2.65 |
|  | Pucciarelli |  |  | 1.08 | 1.02 | 1.13 |
| **Functional Status/Cohabitation** | Almussallam | Alone | Not Alone | 1.00 | 0.70 | 1.20 |
|  | Devon | No | Yes | 1.21 | 1.03 | 1.43 |
|  | Kariv (Continuous variable) | 2.22 | 2.05 | 2.02 | 1.15 | 3.56 |
| **Sex** | Almussallam | Male | Female | 1.00 | 0.80 | 1.30 |
|  | Barina |  |  | 0.79 | 0.73 | 0.87 |
|  | Bliss |  |  | 0.99 | 0.95 | 1.03 |
|  | Damle |  |  | 0.99 | 0.96 | 1.04 |
|  | Devon |  |  | 0.87 | 0.78 | 0.96 |
|  | Greenblatt |  |  | 0.80 | 0.71 | 0.88 |
|  | Kulaylat |  |  | 1.09 | 0.97 | 1.22 |
|  | Lucas |  |  | 0.88 | 0.83 | 0.93 |
|  | Lumpkin |  |  | 0.95 | 0.93 | 0.97 |
|  | Pucciarelli |  |  | 0.84 | 0.82 | 0.87 |
|  | Rattan |  |  | 1.02 | 0.96 | 1.08 |
|  | Schneider |  |  | 1.14 | 1.11 | 1.17 |
|  | Sutton |  |  | 1.19 | 1.03 | 1.37 |
|  | Wick |  |  | 1.04 | 0.98 | 1.11 |
| **Hospital bed size** | Lumpkin | Large | Small | 0.98 | 0.95 | 1.02 |
|  |  |  | Medium | 0.96 | 0.93 | 0.98 |
|  | Rattan | Large | Small | 0.95 | 0.87 | 1.04 |
|  |  |  | Medium | 0.92 | 0.86 | 0.99 |
| **Hospital Volume** | Bliss | Low | Medium | 1.04 | 1.00 | 1.09 |
|  |  |  | High | 1.33 | 1.08 | 1.19 |
|  | Damle | Low | Medium | 1.20 | 1.07 | 1.35 |
|  |  |  | High | 1.17 | 1.05 | 1.31 |
|  |  |  | Very High | 1.11 | 1.00 | 1.24 |
|  | Greenblatt | 1^st^ tertile | 2^nd^ tertile | 1.02 | 0.95 | 1.10 |
|  |  |  | 3^rd^ tertile | 1.05 | 0.97 | 1.14 |
|  | Pucciarelli | 1^st^ quartile | 2^nd^ quartile | 1.03 | 0.98 | 1.07 |
|  |  |  | 3^rd^ quartile | 1.00 | 0.95 | 1.05 |
|  |  |  | 4^th^ quartile | 1.04 | 0.99 | 1.09 |
|  | Sutton | Lowest | Low | 0.75 | 0.61 | 0.91 |
|  |  |  | Medium | 0.70 | 0.58 | 0.85 |
|  |  |  | High | 0.61 | 0.49 | 1.75 |
|  | Rattan | Low | High | 1.04 | 0.96 | 1.11 |
|  | Greenblatt | No | Yes | 1.26 | 1.18 | 1.36 |
| **Hospitalisation year prior to surgery** | Barina | None | One | 1.13 | 1.01 | 1.26 |
|  |  |  | More than one | 1.28 | 1.09 | 1.51 |
|  | Pucciarelli |  |  | 1.23 | 1.19 | 1.27 |
| **Insurance** | Bliss | Private | Medicare | 1.36 | 1.27 | 1.45 |
|  |  |  | Medicaid | 1.52 | 1.39 | 1.65 |
|  | Damle |  | Non-private | 1.28 | 1.22 | 1.35 |
|  | Lumpkin |  | Public | 1.34 | 1.30 | 1.38 |
|  | Rattan |  | Medicare | 1.46 | 1.33 | 1.59 |
|  |  |  | Medicare | 1.76 | 1.58 | 1.95 |
|  | Sutton |  | Medicare/Medicaid | 1.39 | 1.15 | 1.62 |
| **Race** | Bliss | White | Non-white | 1.10 | 1.06 | 1.15 |
|  | Damle |  | Non-white | 1.09 | 1.03 | 1.14 |
|  | Greenblatt |  | Black | 1.08 | 0.94 | 1.23 |
|  |  |  | Asian | 1.26 | 1.06 | 1.50 |
|  |  |  | Hispanic | 1.13 | 0.94 | 1.36 |
|  |  |  | Other | 1.08 | 0.64 | 1.82 |
|  | Kariv |  | Non-white | NS |  |  |
|  | Kulaylat | White | Black | 1.20 | 0.98 | 1.46 |
|  |  |  | Other | 0.87 | 0.63 | 1.20 |
|  | Lucas |  | Black | 1.24 | 1.11 | 1.39 |
|  |  |  | Asian | 0.85 | 0.69 | 1.04 |
|  |  |  | Hispanic | 0.91 | 0.67 | 1.23 |
| **Socioeconomic status/Income** | Bliss | Upper | Lower | 0.97 | 0.94 | 1.01 |
|  | Ghirimoldi | Low | Medium | 2.15 | 1.17 | 4.09 |
|  |  |  | High | 1.88 | 1.08 | 3.40 |
|  | Lumpkin | Highest | Lowest | 1.08 | 1.04 | 1.11 |
|  |  |  | Medium | 1.04 | 1.01 | 1.07 |
|  |  |  | High | 1.03 | 1.00 | 1.06 |
|  | Rattan | >$64000 | $48000 - $63999 | 1.05 | 0.97 | 1.14 |
|  |  |  | $38000 - $47999 | 1.13 | 1.05 | 1.23 |
|  |  |  | $1 - $37999 | 1.05 | 0.96 | 1.14 |
| **Transfer** | Lumpkin | No | Yes | 1.08 | 1.00 | 1.18 |
|  | Kulaylat | No | Yes | 0.90 | 0.33 | 1.29 |
| **Surgeon Volume** | Greenblatt | 1^st^ tertile/lowest volume | 2^nd^ tertile | 0.88 | 0.81 | 0.95 |
|  |  |  | 3^rd^ tertile | 0.85 | 0.78 | 0.92 |
|  | Kulaylat | Top quartile | Bottom quartile | 1.24 | 1.05 | 1.48 |
|  |  |  | Second quartile | 1.03 | 0.86 | 1.23 |
|  |  |  | Third quartile | 0.91 | 0.77 | 1.08 |
| **Operative variables** | | | | | | |
| **Stoma** | Almussallam | Stoma | No Stoma | 1.40 | 1.10 | 1.90 |
|  | Barina |  |  | 2.06 | 1.83 | 2.32 |
|  | Bliss |  |  | 1.33 | 1.27 | 1.40 |
|  | Damle |  |  | 1.53 | 1.45 | 1.61 |
|  | Devon |  |  | 1.26 | 1.09 | 1.45 |
|  | Ghirimoldi |  |  | 2.39 | 1.60 | 3.58 |
|  | Greenblatt |  |  | 1.13 | 1.01 | 1.25 |
|  | Kariv |  |  | NS | NR | NR |
|  | Kulaylat |  |  | 2.31 | 1.76 | 3.03 |
|  | Pucciarelli |  |  | 1.68 | 1.62 | 1.74 |
|  | Toneva |  |  | 1.87 | 1.57 | 2.24 |
|  | Wick |  |  | 1.16 | 1.06 | 1.26 |
| **Surgical Indication** | Bliss | Malignancy | Diverticular Disease | 1.00 | 0.94 | 1.06 |
|  |  |  | Ischaemia | 1.47 | 1.30 | 1.65 |
|  |  |  | Volvulus/Obstruction | 1.12 | 1.06 | 1.18 |
|  |  |  | IBD | 1.28 | 1.17 | 1.41 |
|  |  |  | GI Bleed | 1.22 | 1.11 | 1.34 |
|  |  |  | Other | 1.37 | 1.27 | 1.47 |
|  | Damle | Benign | Diverticular Disease | 0.89 | 0.82 | 0.98 |
|  |  |  | IBD | 1.32 | 1.19 | 1.46 |
|  |  |  | Malignancy | 1.00 | 0.92 | 1.10 |
|  | Keller | Colon Cancer | Benign Anorectum | 1.63 | 0.49 | 5.40 |
|  |  |  | Rectal Cancer | 1.28 | 0.64 | 2.55 |
|  |  |  | Anal Cancer | 4.43 | 0.75 | 23.23 |
|  |  |  | Diverticular Disease | 0.94 | 0.42 | 2.25 |
|  |  |  | IBD | 1.96 | 0.89 | 4.35 |
|  |  |  | Dysmotility | 7.81 | 1.97 | 30.99 |
|  |  |  | Rectal Prolapse | 0.98 | 0.17 | 5.18 |
|  |  |  | Other | 0.40 | 0.15 | 1.06 |
|  | Kulaylat | Malignancy | Diverticular Disease | 1.02 | 0.81 | 1.28 |
|  |  |  | Obstruction | 1.27 | 0.99 | 1.62 |
|  |  |  | Ischaemia | 1.89 | 1.29 | 2.76 |
|  |  |  | IBD | 1.85 | 1.35 | 2.53 |
|  |  |  | Functional | 1.88 | 0.98 | 3.62 |
|  |  |  | Other | 1.42 | 1.19 | 1.71 |
|  | Lumpkin | Malignancy | Trauma | 1.88 | 1.48 | 2.38 |
|  |  |  | IBD | 1.64 | 1.56 | 1.71 |
|  |  |  | Diverticular Disease | 0.80 | 0.78 | 0.82 |
|  |  |  | GI Bleed | 1.50 | 1.25 | 1.79 |
|  |  |  | Ischaemia | 1.77 | 1.59 | 1.97 |
|  |  |  | Infection | 1.48 | 1.25 | 1.75 |
|  |  |  | Obstruction/Volvulus | 1.45 | 1.39 | 1.50 |
|  |  |  | Other | 1.29 | 1.25 | 1.34 |
|  | Turina | IBD | Other | 1.84 | 1.17 | 2.93 |
|  | Rattan | Malignancy | Diverticular Disease | 1.35 | 1.20 | 1.53 |
|  |  |  | Ulcerative Colitis | 2.70 | 2.23 | 3.26 |
|  |  |  | Crohn’s Disease | 1.78 | 1.51 | 2.09 |
|  |  |  | Benign Disease | 0.96 | 0.85 | 1.09 |
|  | Wick | Malignancy | Diverticular Disease | 0.76 | 0.69 | 0.83 |
|  |  |  | IBD | 1.08 | 0.94 | 1.24 |
| **Surgical Approach** | Damle | Open | Laparoscopic/Robotic | 0.76 | 0.69 | 0.83 |
|  | Greenblatt |  | Laparoscopic | 1.16 | 0.79 | 1.71 |
|  | Kariv |  | Laparoscopic | NS | NR | NR |
|  | Keller |  | Laparoscopic | 1.03 | 0.58 | 1.83 |
|  |  |  | Conversion to Open | 0.54 | 0.21 | 1.39 |
|  | Kulaylat |  | Laparoscopic | 0.73 | 0.64 | 0.85 |
|  | Pucciarelli |  | Laparoscopic | 0.94 | 0.90 | 0.97 |
|  | Turina |  | Laparoscopic | 1.94 | 1.23 | 3.07 |
|  | Rattan |  | Laparoscopic | 0.93 | 0.88 | 1.00 |
|  |  |  | Robotic | 1.26 | 0.94 | 1.69 |
| **Surgery Type** | Bliss | Sigmoid Colectomy | Right Colectomy | 1.19 | 1.13 | 1.26 |
|  |  |  | Transverse Colectomy | 1.14 | 1.03 | 1.27 |
|  |  |  | Left Colectomy | 1.16 | 1.08 | 1.24 |
|  |  |  | Total Colectomy | 1.84 | 1.66 | 2.04 |
|  |  |  | APR | 1.51 | 1.34 | 1.17 |
|  |  |  | Low AR | 1.30 | 1.21 | 1.40 |
|  | Damle | Proctectomy | Colectomy | 1.27 | 1.21 | 1.35 |
|  | Kulaylat | Proximal Colectomy | Distal Colectomy | 1.04 | 0.89 | 1.21 |
|  |  |  | Total Colectomy | 1.49 | 1.06 | 2.11 |
|  |  |  | Other Colectomy | 1.16 | 0.93 | 1.44 |
|  | Lucas | Right Colectomy | Left Colectomy | 0.96 | 0.86 | 1.06 |
|  |  |  | Sigmoid Colectomy | 0.88 | 0.81 | 0.96 |
|  |  |  | Transverse Colectomy | 0.90 | 0.89 | 1.04 |
|  |  |  | Total Colectomy | 1.82 | 1.48 | 2.22 |
|  |  |  | Other colectomy | 1.23 | 1.08 | 1.40 |
|  |  |  | APR | 1.19 | 1.05 | 1.35 |
|  |  |  | Low AR | 1.04 | 0.94 | 1.15 |
|  |  |  | Hartmann’s Proctectomy | 1.11 | 0.88 | 1.41 |
|  |  |  | Other Proctectomy | 0.95 | 0.72 | 1.25 |
|  | Rattan | Right Colectomy | Transverse Colectomy | 1.11 | 0.96 | 1.27 |
|  |  |  | Left Colectomy | 0.97 | 0.86 | 1.09 |
|  |  |  | Sigmoid Colectomy | 0.77 | 0.70 | 0.84 |
|  |  |  | Total Colectomy | 1.46 | 1.16 | 1.84 |
|  |  |  | Proctectomy | 1.06 | 0.96 | 1.18 |
|  | Toneva | Partial Colectomy | Rectal Resection | 1.22 | 1.04 | 1.43 |
|  |  |  | Total Colectomy | 2.24 | 1.64 | 3.07 |
|  | Wick | NR | Proctectomy/Colectomy | 1.12 | 1.01 | 1.25 |
| **Surgical urgency** | Almussallam | Elective | Urgent/Emergent | 1.30 | 1.00 | 1.60 |
|  | **Barina** |  |  | 1.41 | 1.25 | 1.58 |
|  | **Bliss** |  |  | 0.95 | 0.91 | 0.99 |
|  | Damle |  |  | 0.96 | 0.90 | 1.02 |
|  | **Devon** |  |  | 1.24 | 1.12 | 1.38 |
|  | **Greenblatt** |  |  | 1.14 | 1.05 | 1.24 |
|  | Kariv |  |  | NS | NR | NR |
|  | Keller |  |  | 1.75 | 0.96 | 3.18 |
|  | **Kulaylat** |  |  | 1.26 | 1.11 | 1.27 |
|  | Pucciarelli |  |  | 1.36 | 0.31 | 1.41 |
|  | **Rattan** |  |  | 0.89 | 0.83 | 0.96 |
| **Rectal procedure** | Barina | Colon | Rectum | 1.34 | 1.20 | 1.50 |
|  | Pucciarelli |  |  | 1.28 | 1.24 | 1.33 |
|  | Schneider |  |  | 1.06 | 1.02 | 1.11 |
| **Postoperative variables** | | | | | | |
| **Any Postoperative Complication** | Almussallam | No | Yes | 16.10 | 12.10 | 21.70 |
|  | Damle |  |  | 1.16 | 1.05 | 1.22 |
|  | Ghirimoldi |  |  | 2.81 | 1.77 | 4.45 |
|  | Greenblatt |  |  | 1.42 | 1.23 | 1.65 |
|  | Keller |  |  | 20.36 | 12.19 | 33.99 |
|  | Kulaylat |  |  | 1.46 | 1.28 | 1.67 |
|  | Schneider |  |  | 1.17 | 1.14 | 1.22 |
| **Blood Transfusion** | Greenblatt | No | Yes | 1.14 | 1.06 | 1.24 |
|  | Schneider |  |  | 1.11 | 1.05 | 1.17 |
| **Discharge Disposition** | Almussallam | Home | Home with Support | 1.00 | 0.80 | 1.30 |
|  |  |  | Rehab | 1.10 | 0.70 | 1.50 |
|  | Bliss |  | Non-Home | 1.55 | 1.49 | 1.63 |
|  | Damle |  | Short-term Nursing Facility | 1.63 | 1.49 | 1.76 |
|  |  |  | Rehab | 2.93 | 2.54 | 3.40 |
|  | Greenblatt |  | Short-term Nursing Facility | 1.34 | 1.23 | 1.46 |
|  | Kariv |  | Other Facility | 4.35 | 0.97 | 20.00 |
|  | Pucciarelli |  | Non-Home | 0.95 | 0.87 | 1.04 |
|  | Rattan |  | Short-term HLC | 1.48 | 0.98 | 3.22 |
|  |  |  | Rest Home Level Care | 2.03 | 1.79 | 2.30 |
|  |  |  | Home with Support | 1.73 | 1.59 | 1.87 |
|  | Wick |  | Non-Home | 1.06 | 1.03 | 1.32 |
| **Ileus** | Francis | No | Yes | 1.36 | 0.46 | 3.96 |
|  | Kariv |  |  | NS | NR | NR |
| **ICU Stay** | Damle | No | Yes | 1.36 | 1.26 | 1.44 |
|  | Keller |  |  | 3.16 | 1.19 | 8.39 |
| **Reoperation** | Damle | No | Yes | 1.05 | 0.91 | 1.20 |
|  | Devon |  |  | 2.41 | 1.59 | 3.67 |
| **LOS** | Bliss | <7 days | >7 days | 1.53 | 1.46 | 1.60 |
|  | Damle | <4 days | >4 days | 1.44 | 1.32 | 1.27 |
|  | Greenblatt | 6 - 14 days | <6 days | 0.96 | 0.82 | 1.12 |
|  |  |  | >14 days | 1.33 | 1.23 | 1.45 |
|  | Kariv | NR | NR | NS | NR | NR |
|  | Pucciarelli | 1 - 9 days | 10 - 13 days | 1.13 | 1.08 | 1.18 |
|  |  |  | 14 - 19 days | 1.34 | 1.28 | 1.41 |
|  |  |  | 20+ days | 1.68 | 1.60 | 1.79 |
|  | Rattan | <7 days | >7 days | 1.37 | 1.27 | 1.48 |
|  | Schneider | Per day | Per day | 1.02 | 1.01 | 1.02 |
|  | Wick | <7 days | >7 days | 1.23 | 1.14 | 1.31 |

S4 Table: Statistically significant preoperative variables (individual studies)

| **Author** | **Variable Characteristic** | **Reference level** | **Significance Level** | **Odd’s Ratio** | **Lower 95% CI** | **Upper 95% CI** |
| --- | --- | --- | --- | --- | --- | --- |
| Almussallam | Patient Setting | Outpatient | Inpatient | 2.00 | 1.30 | 3.20 |
| Almussallam | Anxiolytic use | No | Yes | 1.80 | 1.40 | 2.20 |
| Almussallam | Alcohol | No | Yes | 0.70 | 0.60 | 0.90 |
| Devon | Admission from Institution | No | Yes | 1.67 | 1.44 | 1.93 |
| Francis | Preoperative treatment | No | Yes | 4.49 | 1.41 | 14.35 |
| Kariv | COPD | No | Yes | 7.12 | 1.35 | 37.56 |
| Kariv | Previous Anticoagulants | No | Yes | 4.85 | 1.20 | 19.67 |
| Kariv | Previous Steroids | No | Yes | 2.29 | 1.13 | 4.65 |
| Pucciarelli | Abdominal surgery 3 years prior to surgery | No | Yes | 1.08 | 1.02 | 1.13 |
| Toneva | Bowel Prep | No Bowel Prep | Oral Antibiotic | 0.81 | 0.68 | 0.97 |

S5 Table: Statistically significant operative variables (individual studies)

| **Author** | **Variable Characteristic** | **Reference level** | **Significance Level** | **Odd’s Ratio** | **Lower 95% CI** | **Upper 95% CI** |
| --- | --- | --- | --- | --- | --- | --- |
| Devon | Metastasis | No | Yes | 1.36 | 1.19 | 1.55 |
| Kariv | Perioperative Steroids | No | Yes | 2.49 | 1.15 | 5.39 |
| Kariv | Bowel Perforation | No | Yes | 0.28 | 0.09 | 0.85 |
| Turina | Surgery Time (per 30 minutes) | No | Yes | 1.19 | 1.02 | 1.15 |

S6 Table: Statistically significant postoperative variables (individual studies)

| **Author** | **Variable Characteristic** | **Reference level** | **Significance Level** | **Odd’s Ratio** | **Lower 95% CI** | **Upper 95% CI** |
| --- | --- | --- | --- | --- | --- | --- |
| Francis | ERAS Compliance | No | Yes | 0.38 | 0.18 | 0.84 |
| Greenblatt | Tumour Grade | Moderate | Poor | 1.10 | 1.02 | 1.19 |
| Kariv | Steroids on discharge | No | Yes | 3.04 | 1.35 | 6.84 |
| Wick | Surgical Site Infection | No | Yes | 1.18 | 1.08 | 1.18 |

S7 Table: Statistically non-significant variables (individual studies)

| **Author** | **Variable Characteristic** |
| --- | --- |
| **Preoperative Variables** | |
| Almussallam | Mode of Anaesthesia |
| Almussallam | Smoking |
| Greenblatt | Marital Status |
| Greenblatt | Perforation on Admission |
| Greenblatt | Chemotherapy within 30 days |
| Greenblatt | Hospital setting |
| Kariv | Body Mass Index |
| Kariv | Ischaemic heart Disease |
| Kariv | Congestive Heart Failure |
| Kariv | Diabetes mellitus |
| Kariv | Liver Disease |
| Kariv | Renal Failure |
| Kariv | Cerebrovascular Disease |
| Kariv | Venous Thromboembolism |
| Kariv | Hypertension |
| Kariv | Atrial Fibrillation |
| Kulaylat | Transfer Status |
| Lumpkin | Transferred from another hospital |
| Toneva | Bowel prep |
| Wick | Obesity |
| **Operative Variables** | |
| Almussallam | Anastomosis |
| Kariv | Abscess |
| Kariv | Bowel Obstruction |
| Kariv | Estimated Blood Loss |
| Kariv | Stoma Type |
| Kariv | Anastomosis Type |
| Toneva | Wound Class |
| **Postoperative Variables** | |
| Francis | ERAS Deviation |
| Francis | Discontinuation of intravenous fluids |
| Francis | Epidural anaesthesia failure |
| Greenblatt | Stage |
| Greenblatt | Tumour grade, (reference: moderate, significance level: well-differentiated) |
| Kariv | Nasogastric tube insertion |
| Kariv | Antibiotics on discharge |
| Kariv | Postoperative Fever |
| Kariv | Biochemical test |
| Wick | Emergency Department Visit |
| Hospital/System level variables | |
| Rattan | Hospital Ownership |

#


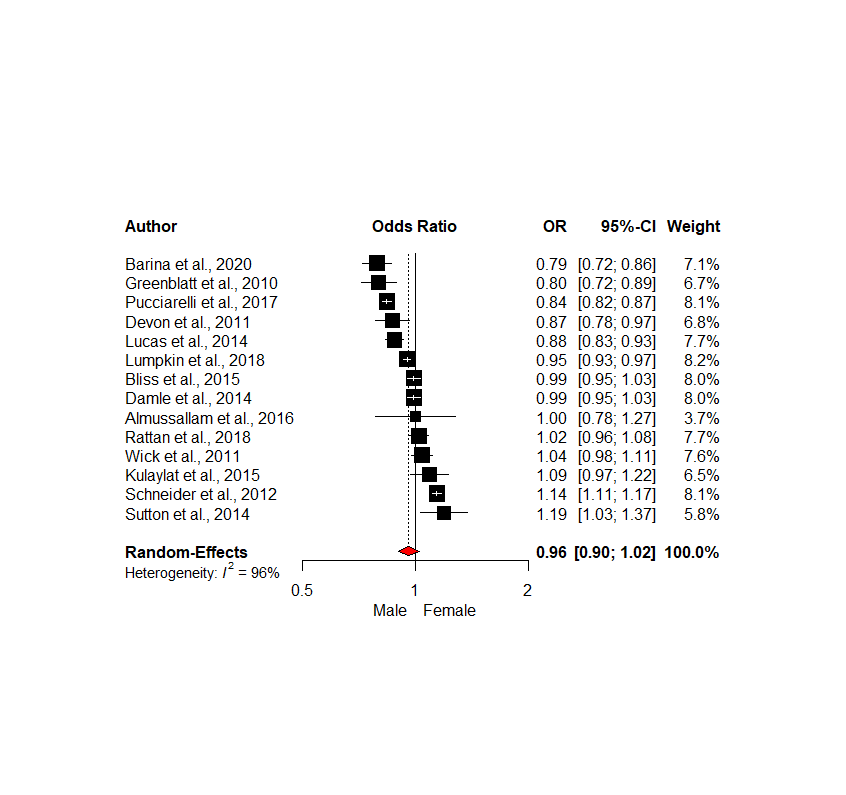


S1 Fig: Forest plot of sex and unplanned readmission


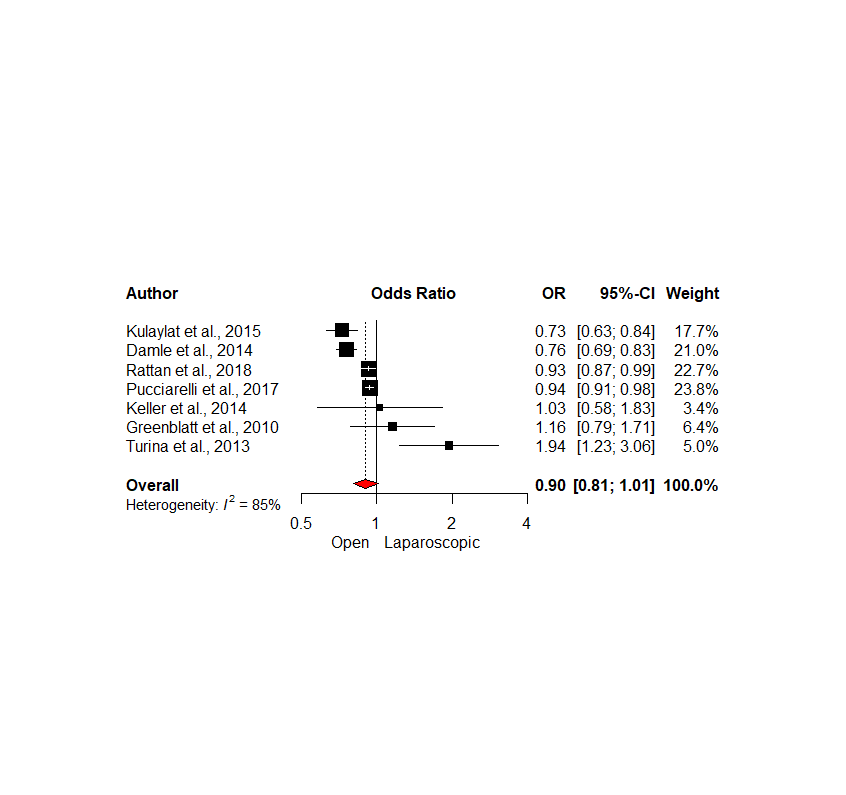


S2 Fig: Forest plot of surgical approach and unplanned readmission

S3 Fig: Meta regression bubble plot (A) Stoma (low vs high risk of bias, p = 0.29), (B) Comorbidity (moderate vs high risk of bias, p = 0.92), and (C) Comorbidity (low vs high risk of bias, p = 0.89)


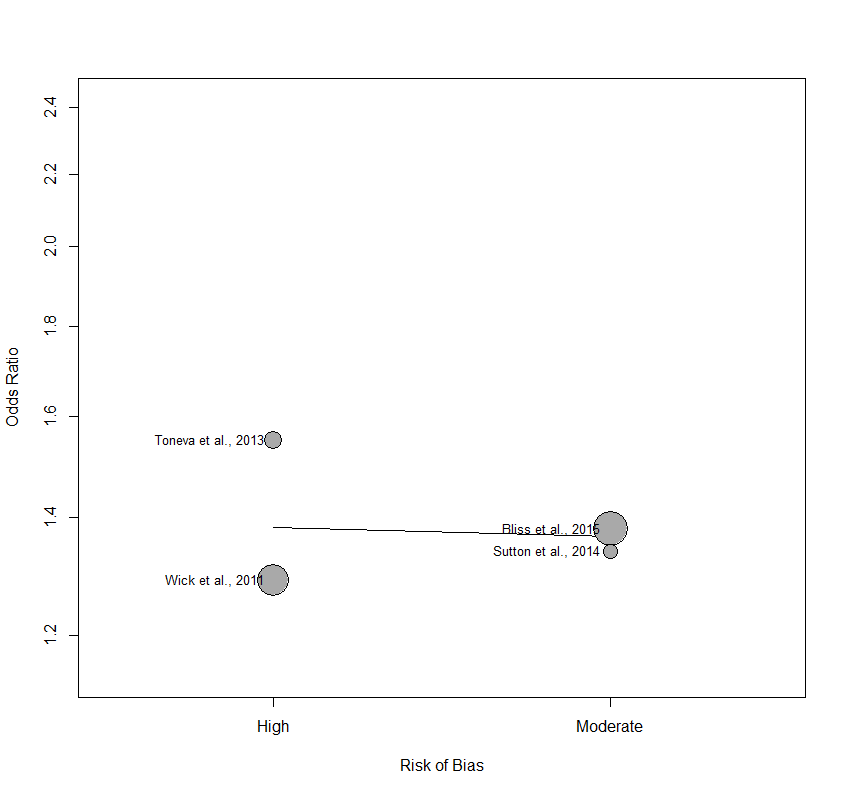

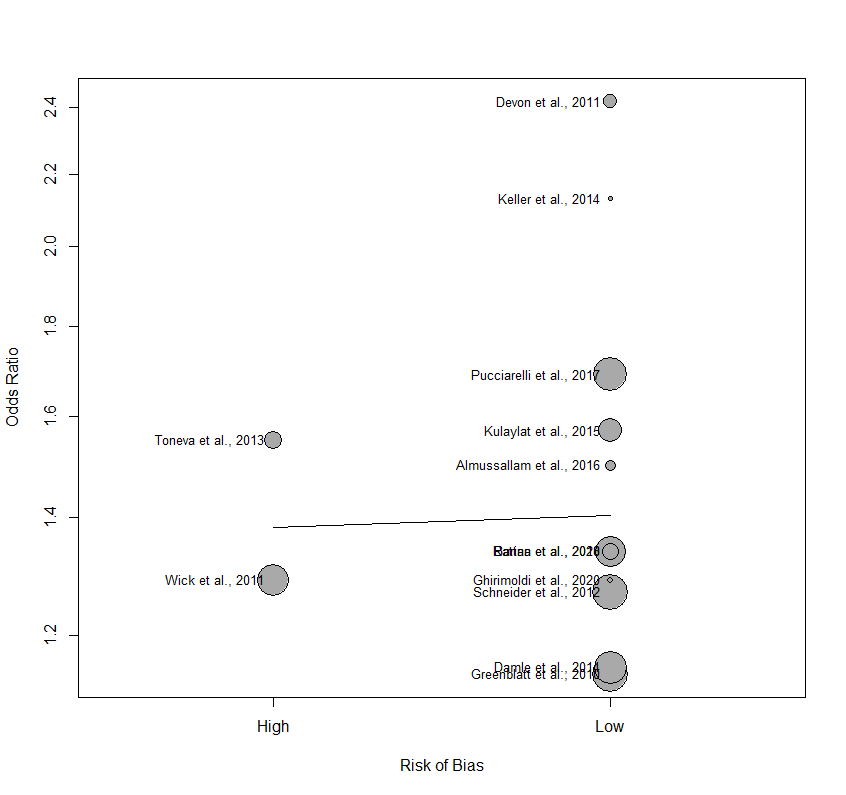

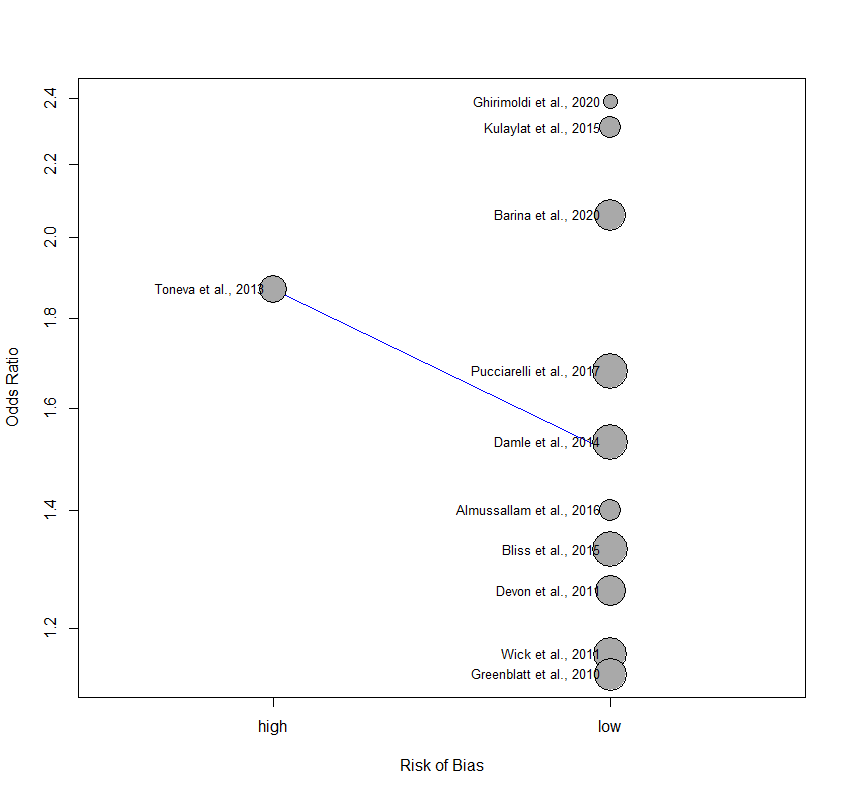


B

A

C


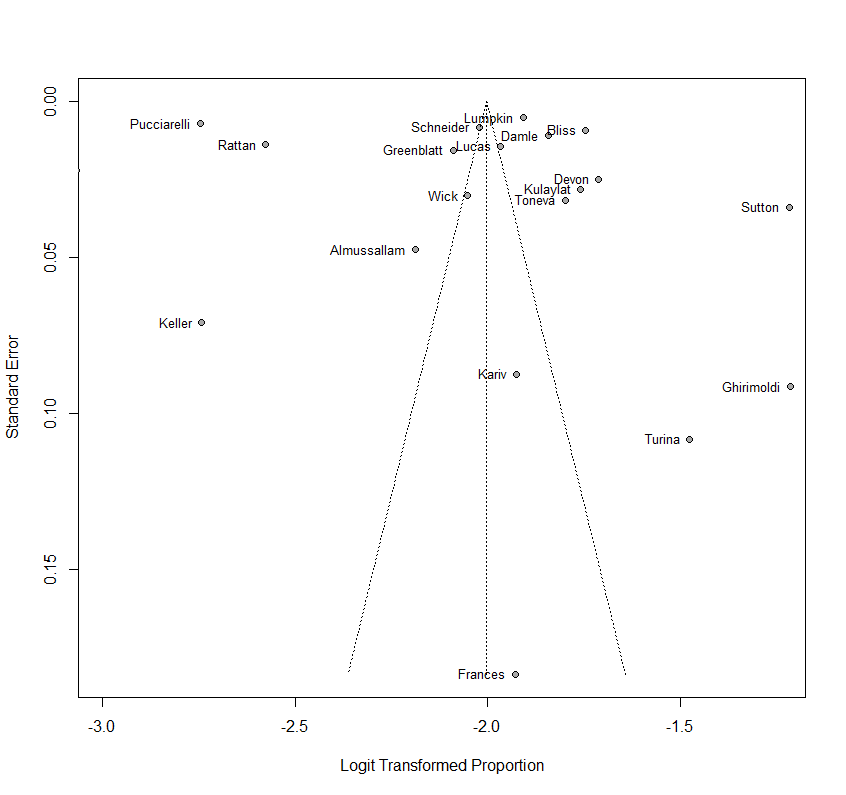


S4 Fig: Funnel plot


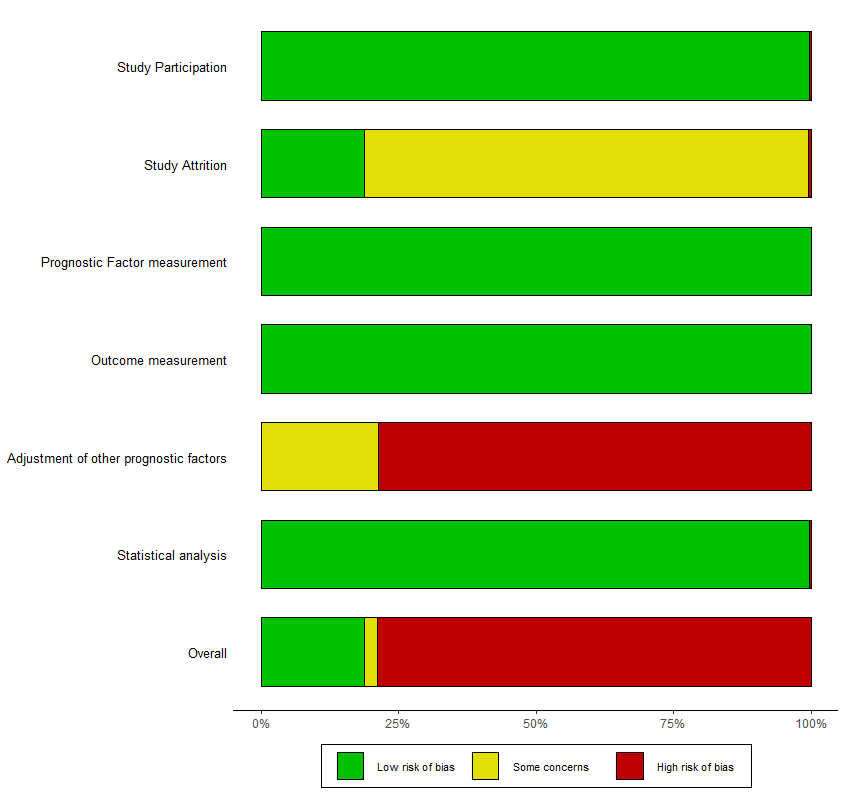


S5 Fig: Risk of bias plot
